# Bone-targeted nanoplatform enables efficient modulation of bone tumor microenvironment for prostate cancer bone metastasis treatment

**DOI:** 10.1080/10717544.2022.2050845

**Published:** 2022-03-14

**Authors:** Xiangyu Zhang, Qingbin Liu, Tingting Zhang, Pei Gao, Hui Wang, Lu Yao, Jingwen Huang, Shulong Jiang

**Affiliations:** a Postdoctoral of Shandong University of Traditional Chinese Medicine, Jinan, China; b Department of Pathology, Jining No. 1 People's Hospital, Jining Medical University, Jining, China; c Clinical Medical Laboratory Center, Jining No. 1 People's Hospital, Jining Medical University, Jining, China; d Jining No. 1 People's Hospital, Jining Medical University, Jining, China; e The First Affiliated Hospital of Bengbu Medical College, Tumor Hospital Affiliated to Bengbu Medical College, Bengbu, China

**Keywords:** Prostate cancer, bone metastasis, calcium phosphate nanoparticle, bone microenvironment, sonic hedgehog (SHH) protein

## Abstract

As there is currently no effective therapy for patients with prostate cancer (PCa) bone metastasis, it was stringent to explore the relevant treatment strategies. Actually, the interaction between cancer cells and bone microenvironment plays important role in prostate cancer bone metastasis, especially the Sonic hedgehog protein (SHH) signaling in the bone microenvironment. The SHH promotes osteoblast maturation and osteoblast then secretes RANKL to induce osteoclastogenesis. Herein, this study develops bone-targeting calcium phosphate lipid hybrid nanoparticles (NPs) loaded with docetaxel (DTXL) and SHH siRNA for PCa bone metastasis treatment. For bone targeting purposes, the nanoplatform was modified with alendronate (ALN). (DTXL + siRNA)@NPs-ALN NPs effectively change the bone microenvironment by inhibiting the SHH paracrine and autocrine signaling, enhancing the anti-tumor effects of DTXL. Besides showing good *in vitro* cellular uptake, the NPs-ALN also inhibited tumor growth both *in vitro* and *in vivo* by inducing apoptosis, cell cycle arrest, and autophagy. This DDS comprised of (DTXL + siRNA)-loaded NPs provides an excellent strategy to treat PCa bone metastasis.

## 1. Introduction

Prostate cancer (PCa) has a relatively high incidence worldwide, and despite the fact that the regional and localized disease is not lethal (5-year survival is almost 100%), the 5-year survival rate decreases to 29.8% when the disease become metastatic (Berish et al., 2018; Siegel et al., 2020). PCa is prone to metastasize to bone and often leads to treatment failure, and chemotherapeutic drugs, such as cabazitaxel and abiraterone acetate only extend the patient's survival for 2.4–4.8 months (Wissing et al., 2015). Bone metastasis causes skeletal-related events (such as bone pain, bone fracture, nerve compression, et al.) that impair the patients’ quality of life, it significantly shortens the survival of PCa patients and often contributes to the death of PCa patients (Coleman, 2006). Therefore, it is urgently necessary to develop therapeutic strategies to prevent the development and progression of PCa bone metastasis.

**Scheme 1.**
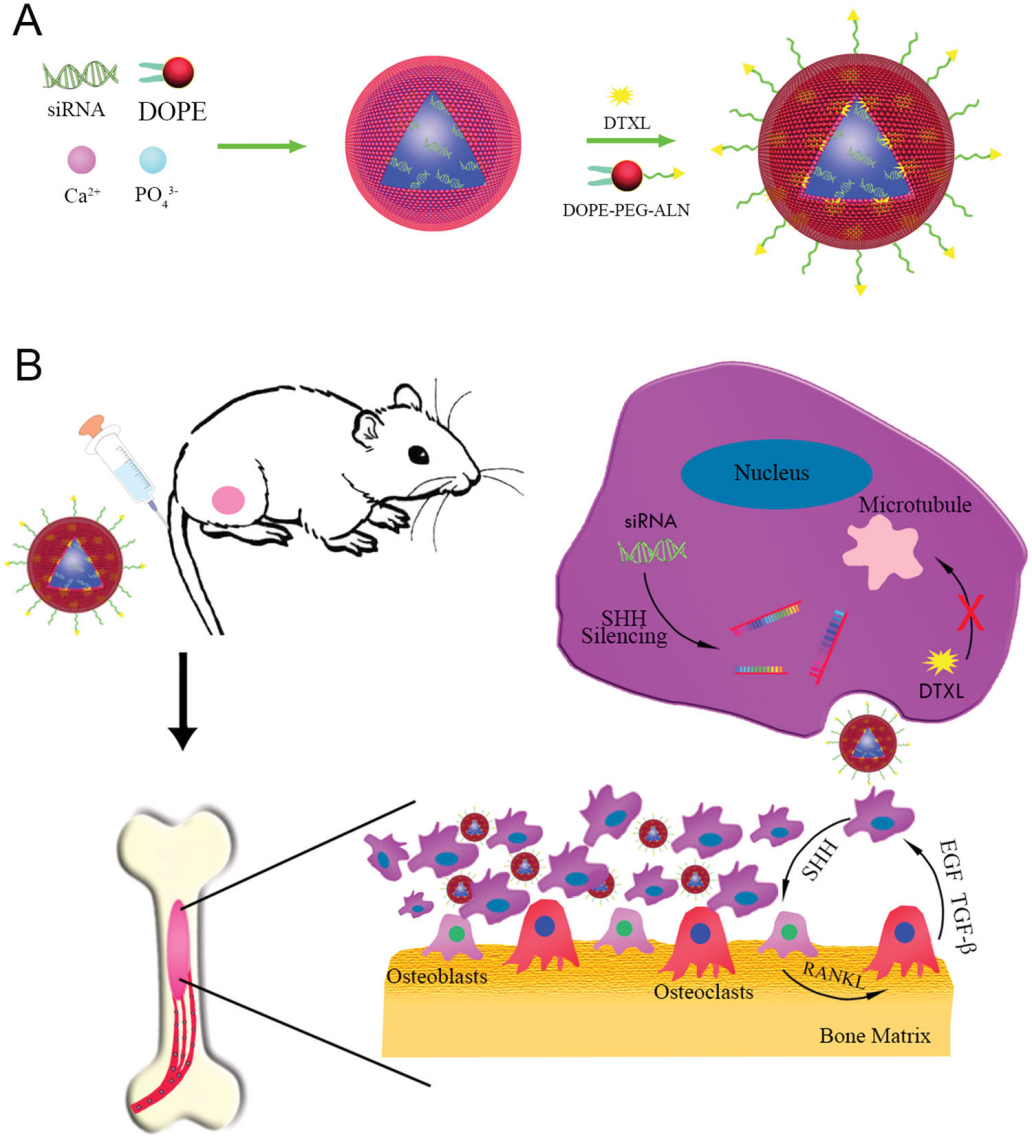
The drug-loaded nanoparticle preparation route (A) and its utilization for PCa bone metastasis treatment (B).

Bone microenvironment plays important role in PCa bone metastasis progression, especially the interaction between bone stromal cells and cancer cells, and we focus on the SHH-RANKL-IL6 signaling network between them (Scheme 1). PCa cells secrete the sonic hedgehog (SHH) protein to promote osteoblast differentiation. SHH binds to the PTCH1 receptor expressed on the cell membrane of osteoblasts and cancer cells, to activate the SHH/PTCH1/GLI1 signaling pathway, which leads to the binding of GLI1 to the promoter of *IL-6* and increases the IL-6 expression (Wu et al., 2017; Zhang, 2019). Additionally, the GLI1 transcription factor promotes the expression of RNAKL, TGF-β, and BMPs (Perrot et al., 2013; Lv et al., 2019). RANKL can activate osteoclastogenesis and promote osteoclasts maturation, which is conducive to cancer cell colonization. Collectively, paracrine SHH signaling effectively promotes PCa bone metastasis. In addition, it has been shown that autocrine SHH signaling potentiates PCa cell proliferation through the SHH-GLI signaling pathway (Sanchez et al., 2004; Ishii et al., 2020). Treatment of cancer cells with the recombinant SHH protein promoted cell proliferation, but blocking SHH signaling with cyclopamine inhibited cancer cell proliferation. Also, treatment of athymic nude mice bearing advanced-stage prostate tumors with cyclopamine and paclitaxel synergistically suppressed the tumor growth, while GLI overexpression could abolish this suppressive effect of cyclopamine (Ang et al., 2017). Additionally, overexpression of GLI was found to confer PCa cells a metastatic phenotype (Wei et al., 2021). Hence, both the SHH paracrine and autocrine signaling pathways were conducive to PCa cell proliferation and bone metastasis.

Nanoparticle-based drug delivery system (DDS) provides a new treatment avenue for PCa bone metastasis. And calcium phosphate (CaP) nanoparticle is one of most used DDS, which possesses good biocompatibility, which is widely used in *in vitro* gene transfection as the ionic interaction between calcium ion and the phosphate group of nucleic acids entails efficient nucleic acid encapsulation, and its pH-responsive biodegradability allows the rapid release of its cargo upon degradation in the acidic lysosomal environment (Kingston et al., 2003). However, the CaP nanoparticle often precipitated rapidly due to its poor colloidal stability, which restricted its treatment effect (Huang et al., 2019). CaP can be functionalized by PEGylation, cationic polymers, natural polymers, cell-penetrating peptides, and biodegradable lipids, and these functionalizations would increase the stability of CaP nanoparticles (Urch et al., 2006; Chen et al., 2016; Chu et al., 2017; Khalifehzadeh & Arami, 2020). The lipid-coated CaP (LCP) is a non-viral gene delivery vehicle, which has been extensively studied by Huang (Li et al., 2012; Tang et al., 2018). In our previous study, we prepared LCP nanoparticle, which was innerly coated with dioleoylphosphatydic acid (DOPA) and outside coated with 1,2-distearoyl-sn-glycero-3-phosphoethanolamine-polyethylene glycol (DSPE-PEG), the double layers of lipid effectively inhibited CaP precipitation (Zhang et al., 2019). Accordingly, LCP NPs have the advantage of both liposomes and CaP. In addition, when the outer layer lipid was modified with PEG, this could prolong the blood circulation time of NPs. For instance, one study encapsulated microRNAs miR-221 and miR-222 into the CaP core, which was then coated with DOPA and encapsulated with PLGA-PEG NPs, this enhanced the *in vivo* gene silencing effects of miRNA-221 and miRNA-222 (Zhou et al., 2017). Additionally, CaP also possesses osteoconductive and osteoinductive characteristics (Pina et al., 2015). siRNA is often used to silence the target gene when it is used for a variety of diseases treatment. However, naked small interfering RNA (siRNA) molecules could be easily degraded by nucleases during blood circulation and the polyanionic properties of siRNA inhibited its endocytosis by cells (Alexis et al., 2008). Therefore, the development of a safe and easily-produced DDS for siRNA loading seems very important. Recently, the CaP NPs have been widely used in RNA interference (RNAi) delivery systems, and coating the CaP NPs with double layers of lipid increased the transfection efficiency of siRNAs, which holds the sustained release manner of the siRNA and longer storage time (Kara et al., 2020; Białas et al., 2021). Hence, we developed a CaP nanoparticle to co-deliver the SHH siRNA and DTXL in this study.

Bisphosphonates have been shown to be able to effectively stabilize CaP NPs without influencing the transfection efficiency, and the bisphosphonate alendronate (ALN) is widely used for NP modification and osteoporosis treatment (Bisso et al., 2019). As ALN binds to hydroxyapatite, which is a major component of bone, it is usually used as a bone-targeting agent for NP modification (Chen et al., 2020; Long et al., 2021). Another important reason for using ALN is that when it binds to CaP, it can inhibit CaP growth. In this study, we used ALN to modify the LCP NPs, to target them to the bone, and achieve their effective delivery and accumulation in the bone metastasis. Docetaxel (DTXL) is used for the treatment of metastatic PCa and is approved by the United States Food and Drug Administration (FDA), and DTXL could effectively prolong the overall survival of metastatic PCa patients. However, DTXL has some adverse side effects, such as hematological toxicity and poor bone penetrability, which limits its antitumor effects. When DTXL is encapsulated into the nanoparticle, its side effects decrease, and the therapeutic effect increase. Therefore, we select the DTXL to treat prostate cancer bone metastasis, and it is loaded into the LCP NPs.

To inhibit prostate cancer bone metastasis progression, we used SHH siRNA to interfere with the SHH-RANKL-IL6 signaling network between bone stromal cells and cancer cells. The bone-targeted LCP NPs was prepared to co-deliver SHH siRNA and DTXL, the CaP in the core of the NPs could absorb the negatively charged siRNA through electrostatic interactions, the CaP core often degrades in the acidic conditions of lysosome to release the siRNA, calcium ions, and phosphate ions, finally the lysosome will burst due to the hyperosmosis caused by calcium ions and phosphate ions. The amphipathic DOPE could encapsulate the lipophilic DTX with high encapsulation efficiency. Additionally, the ALN with a high affinity for bone is an ideal targeted moiety promoting nanoparticles into metastatic PCa cells. Interestingly, this nanoplatform could effectively induce *in vitro* cytotoxicity, cell cycle arrest, and cell apoptosis. In addition, the (SHH siRNA + DTXL) co-delivery nanoplatform effectively inhibited tumor growth and bone destruction of an *in vivo* PCa bone metastasis model. This co-delivery nanoplatform provides a new avenue for PCa bone metastasis treatment.

## 2. Materials and methods

### 2.1. Materials

1,2-dioleoyl-sn-glycero-3-phosphoethanolamine (DOPE), 1,2-dioleoyl-sn-glycero-3-phosphoethanolamine-N-[amino(polyethylene glycol)-2000]-COOH (DOPE-PEG2000-COOH), Cholesterol, disodium hydrogen phosphate (Na_2_HPO_4_), calcium chloride (CaCl_2_), N-(3-dimethylaminopropyl)-N0-ethylcarbodiimide hydrochloride (EDC) and N-hydroxylsuccinimide (NHS) were purchased from Sigma–Aldrich; Docetaxel and alendronate were purchased from MedChemExpress LLC (Monmouth Junction, NJ, USA). The siRNA used in this study was synthesized by Ruibo Biotechnology Inc. (Guangzhou, China). The methyl thiazolyl tetrazolium (MTT) kit, calcein/propidium iodide (PI) cell viability/cytotoxicity assay kit, cell cycle, and apoptosis analysis kit were purchased from Beyotime Biotechnology (Haimen, China). The primary LC3B antibody was purchased from Sigma-Aldrich (Shanghai, China). The primary Bcl-2 antibody and cleaved caspase-3 antibody were obtained from Proteintech Group Inc. (Rosemont, IL, USA); the ERK, p-ERK, GRP78, P62 primary antibodies were purchased from the Cell Signaling Technology Inc. (CST Inc., Danvers, MA, USA). Human SHH enzyme-linked immunosorbent assay (ELISA) kit (ab100639), mouse IL-6 ELISA kit (ab222503), and mouse RANKL ELISA kit (ab100749) were purchased from Abcam (Cambridge, UK). Oligo nucleotide primers used in this study for gene expression level detection were listed in Table 1. The TRAP/ALK stain kit was purchased from Wako (Osaka, Japan). The Alizarin Red S stain kit was purchased from Solarbio (Beijing, China). The LC3-RFP-GFP lentivirus was packaged in our lab according to standard procedure.

**Table 1.** Primers and siRNA sequences.

Name	Species	Forward (5′ to 3′)	Reverse (5′ to 3′)
SHH	Human	GGACAGGCTGATGACTCAGA	GCCCTCGTAGTGCAGAGACT
Ptch1	Mouse	CTCTGGAGCAGATTTCCAAGG	TGCCGCAGTTCTTTTGAATG
SMO	Mouse	CCTGACTTTCTGCGTTGC	GGTTCTGACACTGAATCCC
SUFU	Mouse	GCTTTGAGTTGACGTTTCGT	CAGCATGTGCTGAATTCTTG
Gli1	Mouse	GGAAGTCCTATTCACGCCTTGA	CAACCTTCTTGCTCACACATGTAAG
GAPDH	Human	GTCAGTGGTGGACCTGACCT	TGCTGTAGCCAAATTCGTTG
GAPDH	Mouse	TCCCACTCTTCCACCTTCGATGC	GGGTCTGGGATGGAAATTGTGAGG
		SHH siRNA_001	CCAGAAACTCCGAGCGATT
		SHH siRNA_002	CTCCGAGCGATTTAAGGAA
		SHH siRNA_003	GAAACTCCGAGCGATTTAA

The human PC-3 PCa cell line, mouse MC3T3-E1 cell line, and mouse RAW264.7 cell line were obtained from the Cell Bank of the Chinese Academy of Sciences (Shanghai, China). PC-3 cells were grown in F-12K medium (Gibco; Thermo Fisher Scientific, Inc. Waltham, MA, USA) containing 10% fetal bovine serum (FBS), glutamax, and 1% penicillin/streptomycin (Thermo Fisher Scientific Inc.). MC3T3-E1 and RAW 264.7 cells were maintained in αMEM media (Thermo Fisher Scientific Inc.) and DMEM (Thermo Fisher Scientific Inc.), respectively, supplemented with 10% FBS and 1% penicillin/streptomycin.

The nude male Balb/c mice were used for *in vivo* study, with 4–6 weeks old, body weight ranged from 14 to 21 g, obtained from Jining Medical University (Jining, China). The Animal Care and Use Committee of Jining No. 1 People’s Hospital approved this study (License No. 2018-RM-002).

### 2.2. Synthesis of DOPE-PEG-ALN

The DOPE-PEG-ALN polymer was synthesized according to previous reports (Chu et al., 2017; He et al., 2017). Solution A: 0.063 mmol of DOPE-PEG-COOH (0.063 mmol), EDC and NHS were mixed in 3 mL 2-morpholinoethanesulfonic acid buffer, and vortexed for 60 min.

Solution B: 300 mg of ALN was dissolved in 1 mL NaOH (2 M). Then we added Solution A slowly into solution B under vigorous stirring, then we stirred the mixture for 24 h in a water bath at 37 °C. Then the unreacted monomers and impurities were removed by dialysis (MWCO = 500 Da) against ultrapure water for 3 days, then DOPE-PEG-ALN polymer was collected after freeze-drying. The structure of the DOPE-PEG-ALN was determined using Fourier transform infrared (FTIR) spectroscopy.

### 2.3. *Preparation and characterization of (DTXL* + *siRNA)@NPs-ALN*

Scheme 1 shows the preparation of (DTXL + siRNA)@NPs-ALN. Briefly, Solution A: The CaCl_2_ (300 μL, 500 mM) and siRNA (100 μL, 2 mg/mL) were emulsified in 15 mL cyclohexane. Solution B: The Na_2_HPO_4_ (300 μL, 25 mM, pH 9.0) was dispersed in 15 mL cyclohexane and then mixed with DOPE (200 μL, 20 mg/mL). Then the Solution A and Solution B were mixed and stirred for 45 min, 30 mL of anhydrous ethanol was added, and the mixture was centrifuged to precipitate the CaP cores. The precipitated CaP cores were dissolved in 1 mL of cyclohexane.

Then, 50 μL of DOPE (10 mM)/cholesterol (10 mM) (v:v = 1:1) and 50 μL of DOPE-PEG-ALN (5 mM) with DTXL (DTXL: 0.1 mg/ml) were mixed with the cores, and the oil solvent was evaporated in a vacuum to obtain the lipid membrane. Then, the lipid membrane was suspended in 400 μL Tris-HCl buffer (10 mM, pH = 7.4).

A Zetasizer (Malvern Panalytical Ltd., Malvern, UK) was used to measure the size and zeta potential of the NPs. The morphology of the NPs was characterized by transmission electron microscopy (TEM) on A FEI Tecnai G2 Spirit TEM transmission electron microscope (FEI Company, Hillsboro, OR, USA) was used to observe the morphology of the NPs, after the NPs were negatively stained with uranyl acetate solution.

As for the measurement of drug encapsulation efficiency (EE%) and drug loading (DL%), the drug (DTXL and/or siRNA) loaded NPs were filtrated using an ultracentrifugal filter (cutoff 10 kDa, 5000 rpm for 10 min). UV absorbance of DTXL and siRNA was 232 and 259 nm, respectively. And the EE % and DL % was calculated using the following equations:

EE %= (total drug−unloaded drug)/total drug ×100%

DL %=weight of drug in NPs/weight of NPs×100%

### 2.4. Gel-retardation assay

The agarose gel electrophoresis was conducted to observe the siRNA encapsulated in the NPs, and the free siRNA was used as control, the siRNA in the gel was visualized using a gel imaging system (Tanon Science & Technology, Shanghai, China). The electrophoresis was: the voltage was 120 V and the time was 20 min.

### 2.5. Storage stability assay

NPs for co-delivering DTXL and siRNA were stored at different times at 37 °C or room temperature in closed ethylene propylene (EP) tubes and then the NP size was measured. The average size of the particles was measured using a Zetasizer.

### 2.6. *In vitro* drug release assay

DTXL@NPs or siRNA@NPs solution (2 mL) was transferred to a dialysis bag (MWCO 3.5 kDa) and then immersed in the PBS with two different pH values (pH = 7.4 or 5.5) and gently shaken. At the indicated time points, 1 mL solution outside the dialysis bay was drawn for UV spectrophotometry analysis, and 1 mL fresh PBS was simultaneously added. The UV spectrophotometry at a wavelength of 232 nm or 259 nm was DTXL and siRNA, respectively (Figure S1).

**Figure 1.**
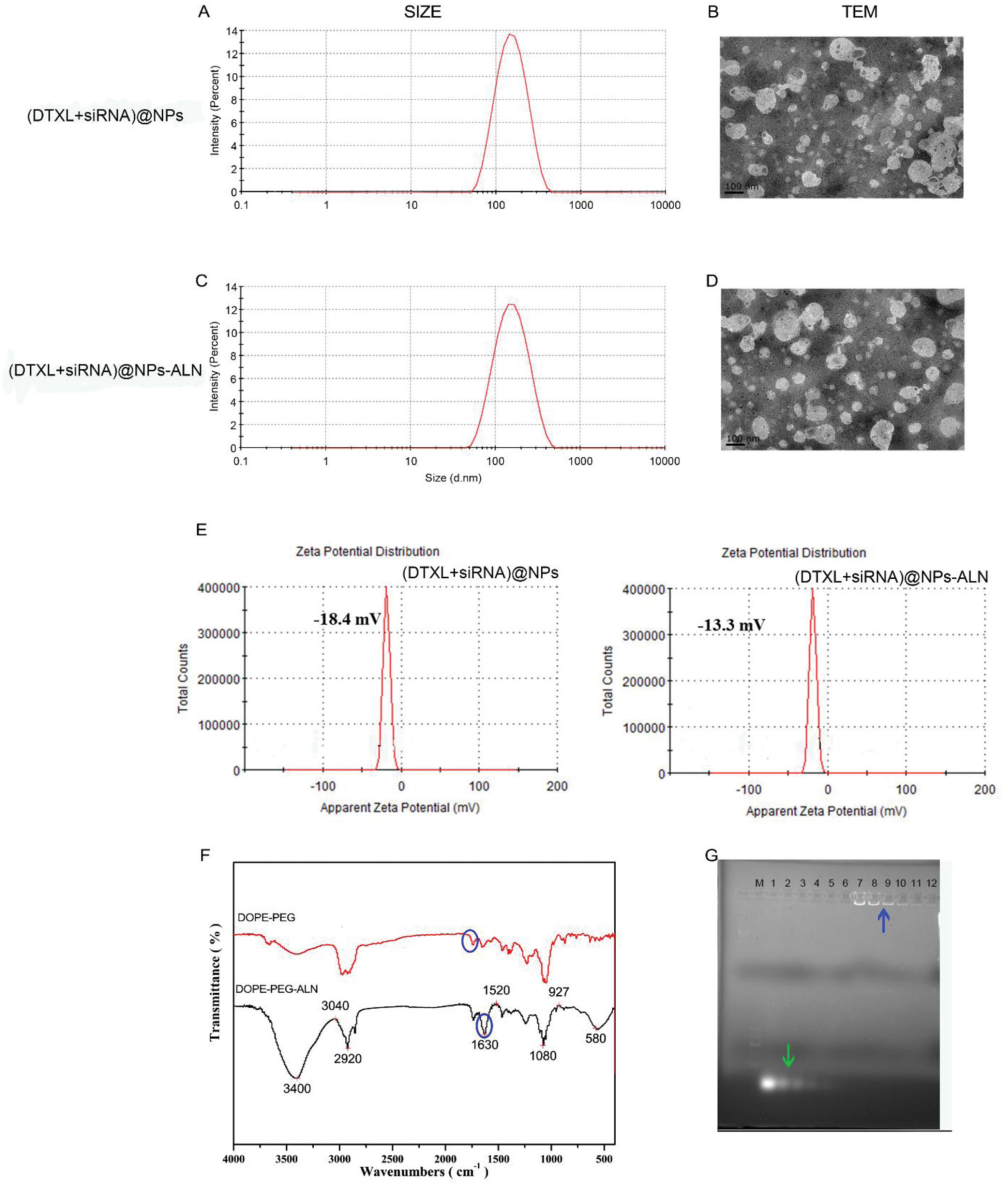
Characterization of the (DTXL + siRNA)@NPs or (DTXL + siRNA)@NPs-ALN nanoparticles. (A) Size distribution of (DTXL + siRNA)@NPs nanoparticle. (B) Transmission electron microscopy (TEM) images of (DTXL + siRNA)@NPs nanoparticle. (C) Size distribution of (DTXL + siRNA)@NPs-ALN nanoparticle. (D) Transmission electron microscopy (TEM) images of (DTXL + siRNA)@NPs-ALN nanoparticle. (E) Zeta potential of (DTXL + siRNA)@NPs and (DTXL + siRNA)@NPs-ALN nanoparticle. (F) FTIR spectra of DOPE-PEG and DOPE-PEG-ALN. (G) The encapsulation capability of (DTXL + siRNA)@NPs-ALN for siRNA using a gel-retardation assay, green arrow indicated the free siRNA and blue arrow indicated the siRNA@NPs.

### 2.7. Thermogravimetric analysis (TGA)

To study the thermal stability of the NPs and (DTXL + siRNA)@NPs, the TGA analysis was conducted. The blank NPs and (DTXL + siRNA)@NPs were analyzed by TGA using Pelkin-Elmer TGA 7 Thermogravimetric analyzer (Perkin-Elmer, Waltham, MA, USA). The temperature ranged from 0 to 800 °C at 10 °C/min of heating rate.

### 2.8. Cell viability assay

PC-3 cells were seeded at 5 × 10^3^ cells/well and incubated with different concentrations of DTXL or siRNA (Table 2) for 24 h. Subsequently, the cells were incubated with 100 μL of MTT-water solution (5 mg/mL) for 4 h. Afterward, 100 μL of DMSO was added after discarding the culture medium. The OD value was then measured using a microplate reader at 490 nm. The combination index (CI) of DTXL and siRNA was calculated using Chou-Talalay combination index equation. The PC-3 cells proliferation rate was assessed after incubation with free DTXL or (DTXL + siRNA) loaded NPs for a different time.

**Table 2.** The drug combination used for killing PC-3 cells.

DTXL	Concentration (nM)	siRNA	Concentration (nM)	Inhibition rate
DTXL	9			0.06
DTXL	18			0.17
DTXL	36			0.50
DTXL	72			0.70
DTXL	144			0.97
		siRNA	12.5	0.00001
		siRNA	25	0.00001
		siRNA	50	0.00001
		siRNA	100	0.00001
		siRNA	100	0.00001
DTXL (18 nM) + siRNA (25 nM)				0.28
DTXL (36 nM) + siRNA (50 nM)				0.58
DTXL (36 nM) + siRNA (50 nM)				0.89

With respect to calcein AM/PI staining for detecting cell viability, PC-3 cell was treated with different drug formulations for 24 h, then 500 µL of PBS containing calcein AM and PI was added. Subsequently, the live or dead cell was observed using a fluorescence microscope.

### 2.9. In vitro cellular uptake

As described above in the 2.3 section of Materials and Methods, we used FAM-siRNA to prepare Solution A, Solution B did not change, then the CaP core was mixed with DOPE/cholesterol and DOPE-PEG (or DOPE-PEG-ALN) to get FAM-siRNA@NPs (or FAM-siRNA@NPs-ALN). After PC-3 cells were seeded at 6-well plates, then free 5(6)-carboxyfluorescein (FAM)-siRNA, and FAM-siRNA@NPs-ALN were incubated with PC-3 cells for 24, 48 h. The excitation (Ex) and emission (Em) wavelengths of FAM are 480 and 525 nm, respectively. Then, the PC-3 cells were washed, trypsinized, and harvested. The uptake of NPs by cells was determined by flow cytometry on a NovoCyte flow cytometer. We also examined the cellular uptake of FAM-siRNA@NPs, and FAM-siRNA@NPs-ALN by confocal laser scanning microscopy (CLSM) using a confocal laser scanning microscope (Olympus Optical Company, Ltd.), with an incubation time of 24 and 48 h.

### 2.10. The SHH, IL-6, and sRANKL protein determination by ELISA

The PC-3 cells were co-cultured with mouse MC3T3-E1 osteoblasts for 24 h, and the supernatant was collected and analyzed for the concentration of SHH, IL-6, and sRANKL using the corresponding ELISA kit according to the manufacturers’ instructions.

### 2.11. Real-time quantitative PCR analysis

As three siRNA against SHH and scrambled siRNA were synthesized and were used for PC-3 cell transfection according to the manufacturer's instructions. The total RNA of the cell was extracted from cells using Trizol reagent and then was reverse transcribed into cDNA. The BeyoFast™ SYBR Green qPCR Mix (2X) (Beyotime Biotechnology) was used to evaluate the target gene (the primers of the target gene are in Table 1) expression level on the Applied Biosystems 7500 Fast Real-Time PCR System. The PCR data were analyzed by 2^−ΔΔCT^ method. The sequences of all primers used are listed in Table 1.

### 2.12. Cell cycle and cell apoptosis analysis

After PC-3 cells were seeded in 6-well culture plates, cells were further treated with different formulations of DTXL or siRNA for 24 h. Subsequently, cells were collected and stained with PI for cell cycle analysis or stained with Annexin V-FITC and PI for cell apoptosis analysis, the analysis was conducted using a NovoCyte flow cytometer.

### 2.13. Western blot analysis

The total protein was extracted from PC-3 cells, and the concentration was measured using a BCA protein assay. And then sodium dodecyl sulfate-polyacrylamide gel electrophoresis (SDS-PAGE) was used to separate the protein and then protein was transferred to nitrocellulose membranes. The membrane was incubated with a corresponding primary antibody and then incubated with a secondary antibody at 37 °C for 1 h, finally imagined using a Tanon 2500 R imaging system.

### 2.14. Trap/ALK stain and alizarin red S stain

For co-cultures of tumor cells with MC3T3-E1 osteoblasts, MC3T3-E1 cells were seeded at 2 × 10^5^ cells/well in 12-well plates. After the cell monolayer reached confluence, PC-3 cells were added at 1 × 10^4^ cells/well in triplicate. Then, the (DTXL + siRNA)@NPs-ALN NPs were added into the co-culture system. After 6 days, the culture media were collected for measurement of osteoblast-secreted IL6 and RANKL protein levels by ELISA, and the cancer cell-secreted SHH protein level was also measured by ELISA.

To determine the co-culture media effect on osteoblast differentiation, the MC3T3-E1 cells were treated with conditioned media, consisting of co-culture media (1:1) mixed with fresh αMEM media (supplemented with ascorbic acid and β-glycerophosphate). With medium replenishment every 3 days, the osteoblast differentiation was assessed after 16 days by staining osteoblasts with Alizarin Red S following the manufacturer’s instructions.

To study the osteoclastogenesis-promoting effect, the pre-osteoclast RAW264.7 cells were seeded in a 6-well plate. Then, the recombinant SHH protein (50 ng/mL) and conditioned media, consisting of co-culture media (1:1) mixed with fresh DMEM media, were added to the RAW264.7 cell. Then, the media was replenished on day 4, and TRAP staining was performed on day 6 according to the manufacturer’s instructions.

### 2.15. Cell autophagy analysis

#### 2.15.1. Transmission electron microscopy (TEM)

First PC-3 cells were treated with different formulations of DTXL and siRNA, then fixed using 2.5% glutaraldehyde. The samples were dehydrated in gradient ethanol and embedded in Epon-Araldite resin. The cell was cut into an ultrathin section and observed using a JEM2100HC transmission electron microscope (Hitachi Ltd., Tokyo, Japan).

#### 2.15.2. mRFP-GFP-LC3 assay

The PC-3 cells were transfected with mRFP-GFP-LC3 lentivirus, then we analyzed the punctum formation using Image Pro Plus 6.0 software. With respect to the autophagy flux assay, the autophagosomes puncta were yellow in merged images, and autolysosomes puncta were red.

### 2.16. Cell migration and invasion analysis

To investigate the effect of drug-loading nanoparticle on PC-3 cell migration and invasion, the transwell chambers (8-μm pores, Corning, NY, USA) was used. For the invasion analysis, the Matrigel (356234; Corning Inc.) was mixed with DMEM at 1:6, then 50 μL of diluted Matrigel was added to the upper chamber and incubated for 1 h at 37 °C, the upper chamber was coated with Matrigel. The PC-3 cells were fixed with glutaraldehyde and stained with crystal violet.

### 2.17. Animal study

For bone metastasis studies, the mouse knee was flexed and then inserted a 27 G needle into the intramedullary canal of the tibia. Then, PC-3 cells (1 × 10^6^ cells, 10 μL) were suspended in 10 μL PBS and then injected into the proximal hole of the right tibia using a 30 G needle, and then the mice are monitored until they fully recover. The proximal tibias and ipsilateral distal femurs which loaded the tumor cells were assessed using a Hiscan XM Micro CT (Suzhou, China). The Animal Care and Use Committee of Jining No. 1 People’s Hospital approved this study (License No. 2018-RM-002).

### 2.18. Antitumor efficacy

On the 4th day after tumor cell injection, the tumor-bearing nude mice (*n* = 6 per group) were injected via tail vein, with PBS, free (DTXL + siRNA), or drug-loaded nanoparticle at the dose of DTXL 2 mg kg^−1^ and siRNA 1.3 mg kg^−1^ every 2 days for 12 times. Micro-computed tomography (micro-CT) imaging was used to assess the volume of bone metastasis. On the 35th day, the mice were sacrificed, tumor volumes were evaluated. Hematoxylin and eosin (H&E) staining and terminal deoxynucleotidyl transferase dUTP nick end labeling (TUNEL) staining were performed to observe the tumor morphology and evaluate tumor apoptosis.

### 2.19. Statistical method

SPSS 16.0 statistical software was used to analyze the relevant data. A one-way analysis of variance (ANOVA) was used to measure the differences between the means of multiple samples. The groups were compared using the Student–Newman–Keuls (SNK) method, and *p* < .05 was considered to be statistically significant.

## 3. Results

### 3.1. *Characterization of (DTXL* + *siRNA)@NPs-ALN*

As shown in Scheme 1, we prepared the NPs loaded with DTXL or siRNA. The (DTXL + siRNA)@NPs were 160.6 nm in size (Figure 1(A)), and their zeta potential was −18.4 mV (Figure 1(E)), while the size of (DTXL + siRNA)@NPs-ALN was 166 nm (Figure 1(C)), and their zeta potential was −13.3 mV (Figure 1(E)), which indicated that the targeting moiety did not significantly increase the size of the NPs, but slightly increase their zeta potential. Additionally, the FTIR spectra result indicated the –C = O signal of PEG was ∼1730 cm^−1^, and when the PEG was modified with ALN, the –NH–C = O signal was 1640 cm^−1^ (Figure 1(G)). Thus, we can surmise that most of the –COOH on PEG-COOH participated in the amidation reaction with ALN. When we observed the morphology of NPs using TEM, it was found that NPs surface was smooth with no apparent aggregation (Figures 1(B,D)). As the NP was used to load the siRNA, we used the Gel retardation method to analyze the siRNA loading capacity, in which the free siRNA moved in the agar gel under the applied electric field (lane 1–6, Figure 1(H)), while the siRNA encapsulated in the NP cannot move in the agar gel (lane 7–12, Figure 1(H)).

### 3.2. NP stability and drug release profile

#### 3.2.1. Storage stability assay

**Figure 2.**
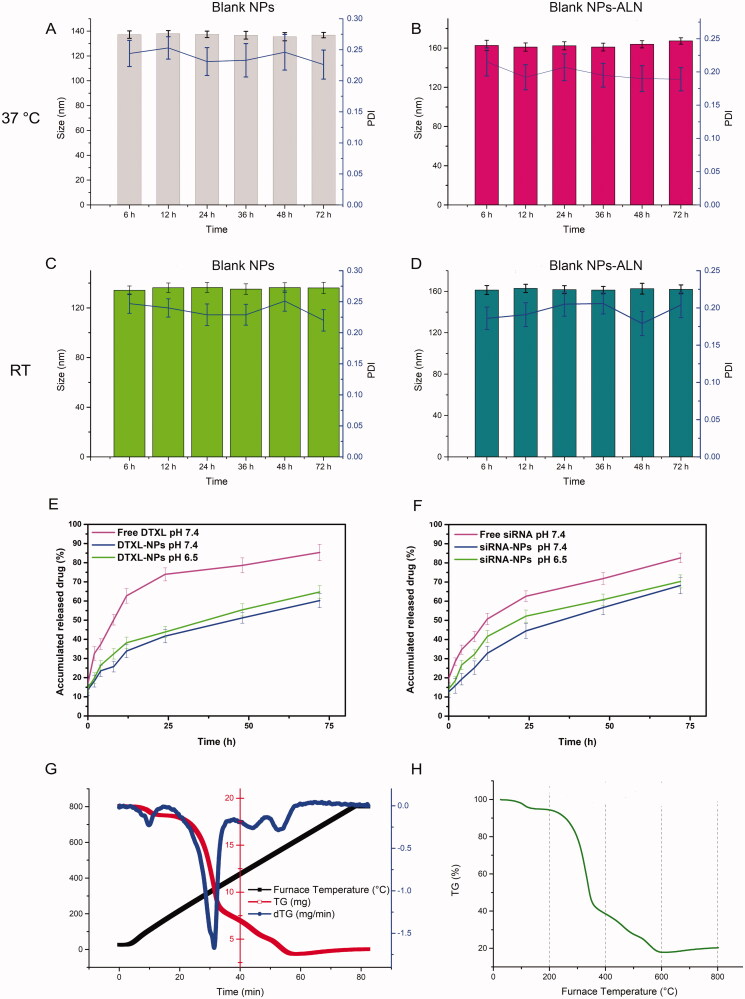
Stability, drug release profile and thermogravimetric analysis of the nanoparticle. (A&B): Size of (DTXL + siRNA)@NPs and (DTXL + siRNA)@NPs–ALN nanoparticle in PBS at 37 °C. (C&D): Size of (DTXL + siRNA)@NPs and (DTXL + siRNA)@NPs-ALN nanoparticle in PBS at room temperature. (E) DTXL released from (DTXL + siRNA)@NPs-ALN nanoparticles in PBS. (F) siRNA released from (DTXL + siRNA)@NPs-ALN nanoparticles in PBS. (G) thermogravimetric analysis of (DTXL + siRNA)@NPs nanoparticle. (H) thermogravimetric analysis of (DTXL + siRNA)@NPs-ALN nanoparticle.

Our evaluation of the stability of NPs at 37 °C or room temperature revealed that, at 37 °C, the size of the blank NPs ranged from 135.5 to 138 nm, and their PDI ranged from 0.22 to 0.25 (Figure 2(A)), while the size of the blank NPs-ALN ranged from 161.1 to 167.3 nm, and their PDI ranged from 0.18 to 0.21 (Figure 2(B)). The size of drug-loaded NPs was relatively stable, and they did not aggregate during the 72 h at 37 °C. When we evaluated NPs stability at room temperature, the size of the blank NPs ranged from 134.2 to 136.5 nm, and their PDI ranged from 0.22 to 0.25 (Figure 2(C)), while the size of the bank NPs-ALN ranged from 161.3 to 162.9 nm, and their PDI ranged from 0.17 to 0.20 (Figure 2(D)). The size and PDI of (DTXL + siRNA)@NPs or (DTXL + siRNA)@NPs-ALN showed no obvious change within 72 h at 37 °C or room temperature, possessing good stability.

#### 3.2.2. Drug release profile

We evaluated the drug encapsulation efficiency and drug loading efficiency of (DTXL + siRNA)@NPs-ALN. The results indicated that the drug encapsulation efficiency of DTXL and siRNA was 96.7 and 84.9%, respectively. And the drug loading efficiency of DTXL and siRNA was 5.3 and 6.3%, respectively.

The evaluation of the drug release profile of the DTXL@NPs or siRNA@NPs revealed that DTXL or siRNA were released in a controlled manner, which was favorable for exerting sustainable anti-tumor effects. The drug release profile of DTXL or siRNA from NPs was evaluated at both pH 7.4 and pH 6.5. With respect to DTXL, free DTXL released 73.9% at 24 h at pH 7.4, while the DTXL@NPs released DTXL 41.6% at pH7.4 and 43.8% within the first 24 h. The DTXL release profile did not change much when the pH value decreased from 7.4 to 6.5. When DTXL was loaded into NPs, it released much slowly, and the pH did not influence DTXL release obviously (Figure 2(E)). And as for siRNA, free siRNA released 63.2% at 24 h at pH 7.4, while the siRNA@NPs released siRNA relatively slowly within the first 24 h (44.5%) at pH 7.4, and 52.2% at pH 6.5. This was possible due to disaggregate of calcium phosphate core of NPs, as the siRNA was encapsulated in the CaP core of the NPs (Figure 2(F)).

#### 3.2.3. Thermogravimetric analysis (TGA)

The weight loss of (DTXL + siRNA)@NPs and (DTXL + siRNA)@NPs-ALN began at 30 °C, the loss rate was about 5%, this is the first stage; the second weight loss stage ranged from 300 to 400 °C, which was much rapid and weight loss rate was 85%; the final stage ranged from 400 to 800 °C, the weight loss rate was around 10%. These results indicated that the drug/siRNA, phospholipid, and other organic compounds are at a high rate at around 90% (Figures 2(G,H)).

### 3.3. *In vitro combinational anti-tumor effects of (DTXL* + *siRNA)@NPs-ALN*

The IC_50_ values of different formulations DTX showed that the IC_50_ of free DTXL was 36.2 nM. However, the IC_50_ of DTXL in the free (DTXL + siRNA) treatment was 24.5 nM, while in the (DTXL + siRNA)@NPs-ALN treatment, the IC_50_ of DTXL was 12.8 nM. The CI value of DTXL (36.2 nM) and siRNA (50 nM) was 0.85, this indicated synergistic effect existed between DTXL and SHH siRNA (Figure S3). These results indicated that SHH silencing sensitizes DTXL cytotoxicity against PC-3 cells, and when SHH siRNA and DTXL were co-delivered using NPs, they possessed enhanced chemotherapeutic effects.

**Figure 3.**
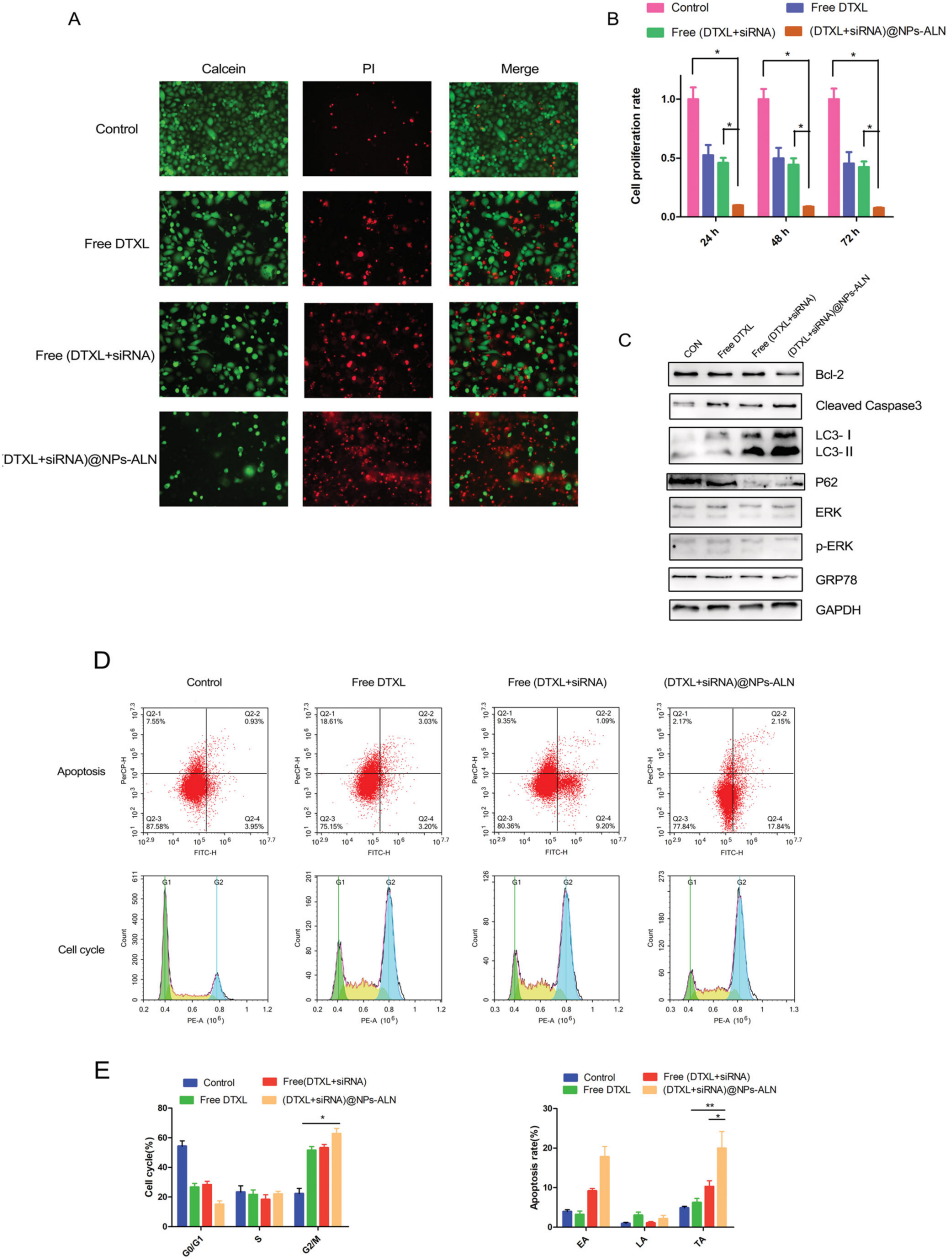
*In vitro* anti-tumor ability of (DTXL + siRNA)@NPs-ALN. (A) Calcein AM and PI staining of PC-3 cells for live and dead cell visualization. (B) cell viability of PC-3 cells treated with different formulation of DTXL and siRNA. (C) Western blotting analysis of relevant protein associated with apoptosis, proliferation and autophagy. (D) Cell cycle and apoptosis analysis after cells incubation with different formulation of DTXL and siRNA. The (DTXL + siRNA)@NPs-ALN group can induce cell apoptosis and cell cycle arrest at G2/M phase. (E) The proportions of G2/M phase cells and the proportion of early apoptosis (EA), the late apoptosis (LA) and total apoptosis (TA) are the most in the (DTXL + siRNA)@NPs-ALN group. Data were expressed as the mean ± *SD* (*n* = 3). *p < .05, **p < .01.

Therefore, we used an equivalent concentration of DTXL (40 nM) and siRNA (50 nM) to treat PC-3 cells to evaluate the cytotoxicity of different formulations of DTXL or siRNA.

The results of the calcein-AM/PI double staining analyses also indicated that tumor cells were effectively killed by (DTXL + siRNA)@NPs-ALN, there were the most dead cells in this group when compared with the free (DTXL + siRNA) group (Figure 3(A)).

We evaluated cell proliferation rate at three time points (24, 48, and 72 h) using the MTT method. When compared with the control group, the (DTXL + siRNA)@NPs-ALN group possessed the least cell proliferation rate (Figure 3(B)). In support of these results, the cleaved caspase 3 expression levels were increased, while the BCL-2 and phosphorylated ERK (p-ERK) were downregulated (Figure 3(C)).

After the PC-3 cells are treated with different formulations of DTXL or siRNA. (DTXL + siRNA)@NPs-ALN effectively induced G2/M phase arrest, the proportion of these phase cells was 62.8%, while the control group, free DTXL group, free (DTXL + siRNA) group was 22.3, 51.6, and 53.2%, respectively (Figures 3(D,E)). In addition, the total apoptosis rate of cells in (DTXL + siRNA)@NPs-ALN group was 19.9%, which was the most; while the total apoptosis of the control group, free DTXL group, free (DTXL + siRNA) group was 4.8, 6.23, and 10.29%, respectively (Figures 3(D,F)).

### 3.4. In vitro cellular uptake

**Figure 4.**
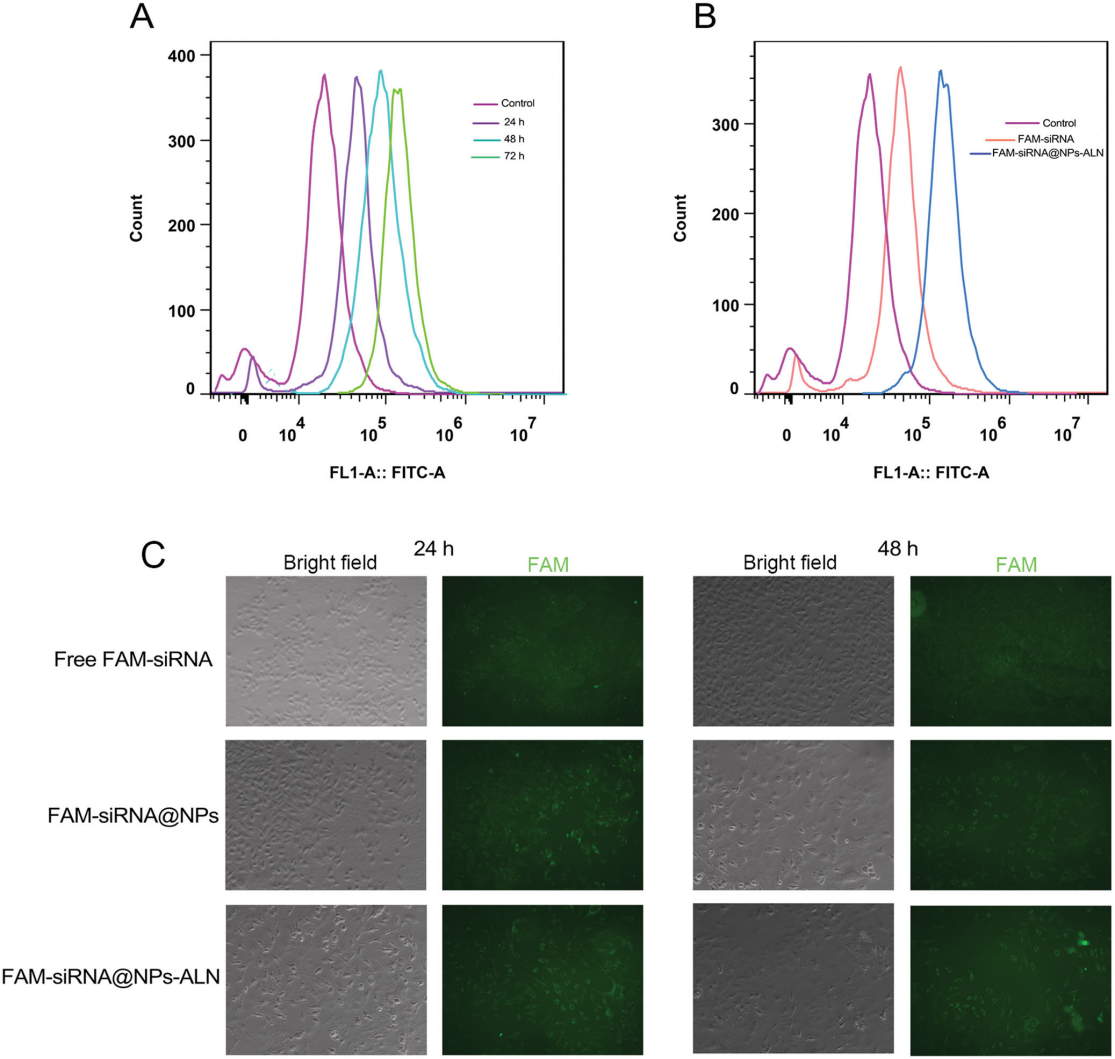
Cellular uptake of FAM-siRNA@NPs-ALN. (A) flow cytometry analysis of FAM fluorescence intensity in PC-3 cells after incubation with nanoparticles for different time points. (B) flow cytometry analysis of FAM fluorescence intensity in PC-3 cells after incubation with different types nanoparticles. (C) CLSM images of PC-3 cells cultured with nanoparticles for different time points.

*In vitro* cellular uptake is important for NPs to exert their antitumor effect. Therefore, both flow cytometry and CLSM were used to examine the effect of NP cellular uptake. Firstly, with the increase of the incubation time, the tumor cells took up more FAM-siRNA@NPs-ALN, and the amount taken up at the 72 h time point was much greater than that at 48 or 24 h (Figure 4(A)). Secondly, the FAM-siRNA@NPs-ALN was internalized much more efficiently than free FAM-siRNA (Figure 4(B)). Lastly, the CLSM results also supported that the FAM-siRNA@NPs-ALN was internalized the most at 48 h compared with the free FAM-siRNA or FAM-siRNA@NPs (Figure 4(C)).

### 3.5. Inhibition of SHH signaling of osteoblast and interreference of osteoclast maturation

**Figure 5.**
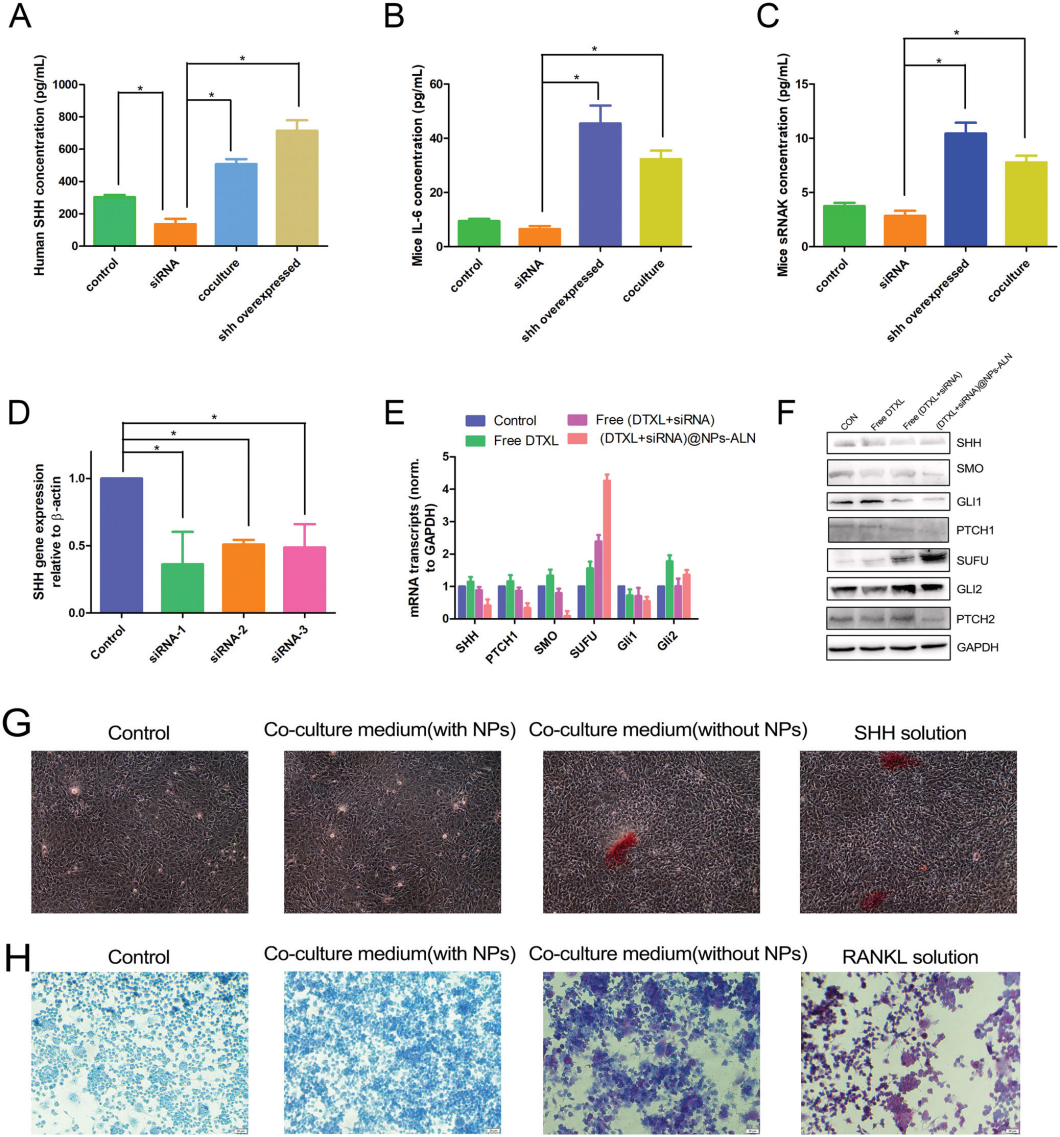
PC-3 cell co-culture with MC3T3-E1 cells. (A) The human SHH protein secretion in co-culture media determined by ELISA. (B) The mice IL-6 protein secretion in co-culture media determined by ELISA. (C) The mice sRANKL protein secretion in co-culture media determined by ELISA. (D) The gene silencing effect of siRNA determined by real-time qPCR. (E) The mice PTCH1, SMO, SUFU, Gli1, and Gli2 mRNA expression level determined by real-time qPCR. (F) The human SHH, PTCH1, SMO, SUFU, Gli1, and Gli2 protein expression level determined by Western blotting. (G) The co-culture media promoted MC3T3-E1 cells differentiation into osteoblast and promoted mineralization. (G) The co-culture media promoted RAW264.7 cells differentiation into osteoclast. Data were expressed as the mean ± *SD* (*n* = 3). *p < .05.

With the aim to study the interplay between PC-3 cells and mice MC3T3-E1 cells, the two cells were co-cultured for 24 h, and then the human SHH protein and mouse IL-6 and sRANKL proteins were analyzed by ELISA. The SHH content in the co-culture group was 507.6 pg/mL, which was much higher than that in the control and siRNA-treated group (*p* < .05). The SHH overexpression group (transfected with SHH lentivirus), which was used as the positive control, was 714 pg/mL (Figure 5(A)). This result was similar to those of the mouse IL-6 and sRANKL proteins, IL-6 and sRANKL in the co-culture group was 32.3 and 10.4 pg/mL, respectively, which was much higher than those in the control and siRNA group (*p* < .05) (Figures 5(B,C)). This was probably due to that co-culture stimulates the PC-3 cells to secrete SHH protein, and this protein induces mouse MC3T3-E1osteoblasts to secrete IL-6 and sRANKL. It was found that the vicious cycle between the cancer cell and osteoblast promoted cancer progression.

Then the (DTXL + siRNA)@NPs-ALN was used to treat PC-3 cells, and then collected the supernatant of PC-3 cells for MC3T3-E1 treatment. We evaluated the expression of SHH signaling pathway-related genes by qRT-PCR, revealing that the PTCH1, SMO, and GLI1 mRNA expression levels in MC3T3-E1cells were decreased while the SUFU mRNA level was increased, and the GLI2 mRNA level was almost unchanged (Figure 5(E)). These results indicated that the paracrine pathway of SHH influenced the expression of the target genes (PTCH1, SMO, Gli1, and SUFU) in MC3T3-E1 cells.

Our evaluation of the SHH autocrine effect on PC-3 cells by Western blot analysis indicated that the expression of the SHH, SMO, GLI1, PTCH1, and PTCH2 proteins was decreased, while that of the SUFU protein was increased. Like the qRT-PCR results, the GLI2 protein did not noticeably change (Figure 5(F)). These results indicated that (DTXL + siRNA)@NPs-ALN effectively inhibited the activation of the SHH-GLI1 signaling pathway in PC-3 cells. Together, the (DTXL + siRNA)@NPs-ALN inhibited activation of the SHH-GLI1 signaling pathway in both MC3T3-E1 osteoblasts and PC-3 cells.

As three siRNA against *SHH* and scrambled siRNA were synthesized, qRT-PCR analysis was performed to determine the most effective siRNA against *SHH*, and it was found that the *SHH* siRNA-1 had the most potent silencing effect against *SHH* in PC-3 cell, the control group (scrambled siRNA) did not silence *SHH* gene expression (Figure 5(D)).

We also analyzed the effects of co-culture supernatant (co-culture of PC-3 cell and MC3T3-E1 cell) on osteoblast and osteoclast differentiation. In particular, for osteoblast differentiation, MC3T3-E1 cells were stained with Alizarin Red S after culturing for 16 days with different treatments. The addition of SHH protein (50 ng/mL) induced the MC3T3-E1 differentiation and mineralization. In addition, the co-culture media also had an effect similar to SHH protein, but the co-culture medium of cells treated with (DTXL + siRNA)@NPs-ALN did not have this effect, likely due to the lack of SHH protein in this media (Figure 5(G)).

For osteoclast differentiation, the RAW264.7 pre-osteoclast cells were treated with mouse recombinant RANKL (50 ng/mL) or co-culture media, then the cells were stained with TRAP. It was found that recombinant RNAKL effectively induced osteoclast differentiation, and conditioned media from co-culture of PC-3 cell with MC3T3-E1 also possessed the effect similar to RANKL, which can induce RAW264.7 differentiation to osteoclast, but conditioned media from cells treated with NPs-ALN did not induce osteoclast differentiation (Figure 5(H)).

### 3.6. Tumor cell autophagy

**Figure 6.**
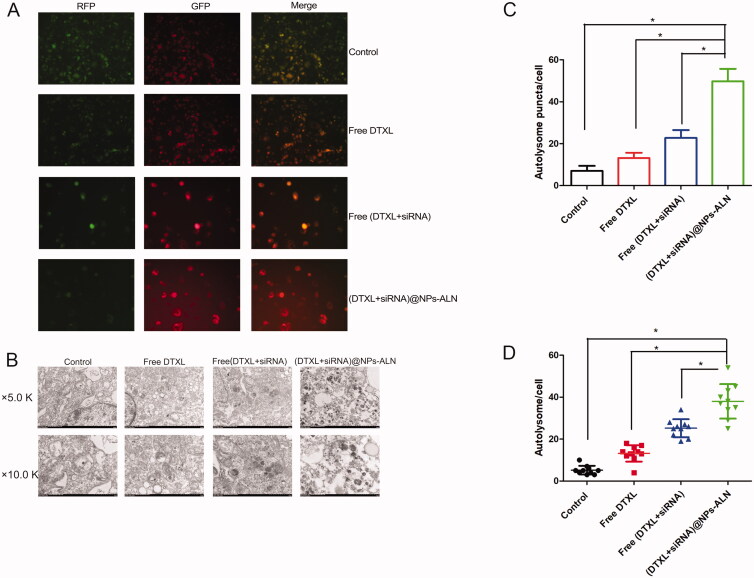
PC-3 cell autophagy analysis after treated with nanoparticles. (A) Fluorescence image indicated the most autolysosome was in the (DTXL + siRNA)@NPs group, while the autophagosomes did not increase significantly. (B) Electron microscopy indicated the most autolysosome and autophagosomes appeared in the (DTXL + siRNA)@NPs-ALN group. (C) Statistics analysis was performed to analyze the number of autolysosome in the cell of different groups, which was observed with fluorescence microscopy; (D) Statistics analysis was performed to analyze the number of autolysosome in the cell of different groups, which was observed with electron microscopy. Data were expressed as the mean ± *SD* (*n* = 3). *p < 0.05.

First of all, PC-3 cells were treated with DTXL or siRNA drugs, then cells were transfected with mRFP-GFP-LC3 lentivirus for 8 h, and the autophagic flux was examined by fluorescence microscopy. As the green fluorescence of the GFP did not appear at the autolysosomes, the red puncta indicate the autolysosome. The (DTXL + siRNA)@NPs-ALN group showed considerably more red puncta compared with other groups, indicating that the autolysosome increased in this group when compared with other groups (Figures 6(A,B)). This result was further confirmed by TEM observation of the autolysosome, which was consistent with the fluorescence microscopy results, revealed that the (DTXL + siRNA)@NPs-ALN treatment dramatically increased the number of autolysosomes (Figures 6(C,D)). Consistent with the fluorescence results and TEM results, the LC3-II was upregulated and the p62 protein was downregulated in the (DTXL + siRNA)@NPs-ALN group, autophagy was effectively induced in this group (Figure 3(C)).

### 3.7. Tumor cell migration and invasion

**Figure 7.**
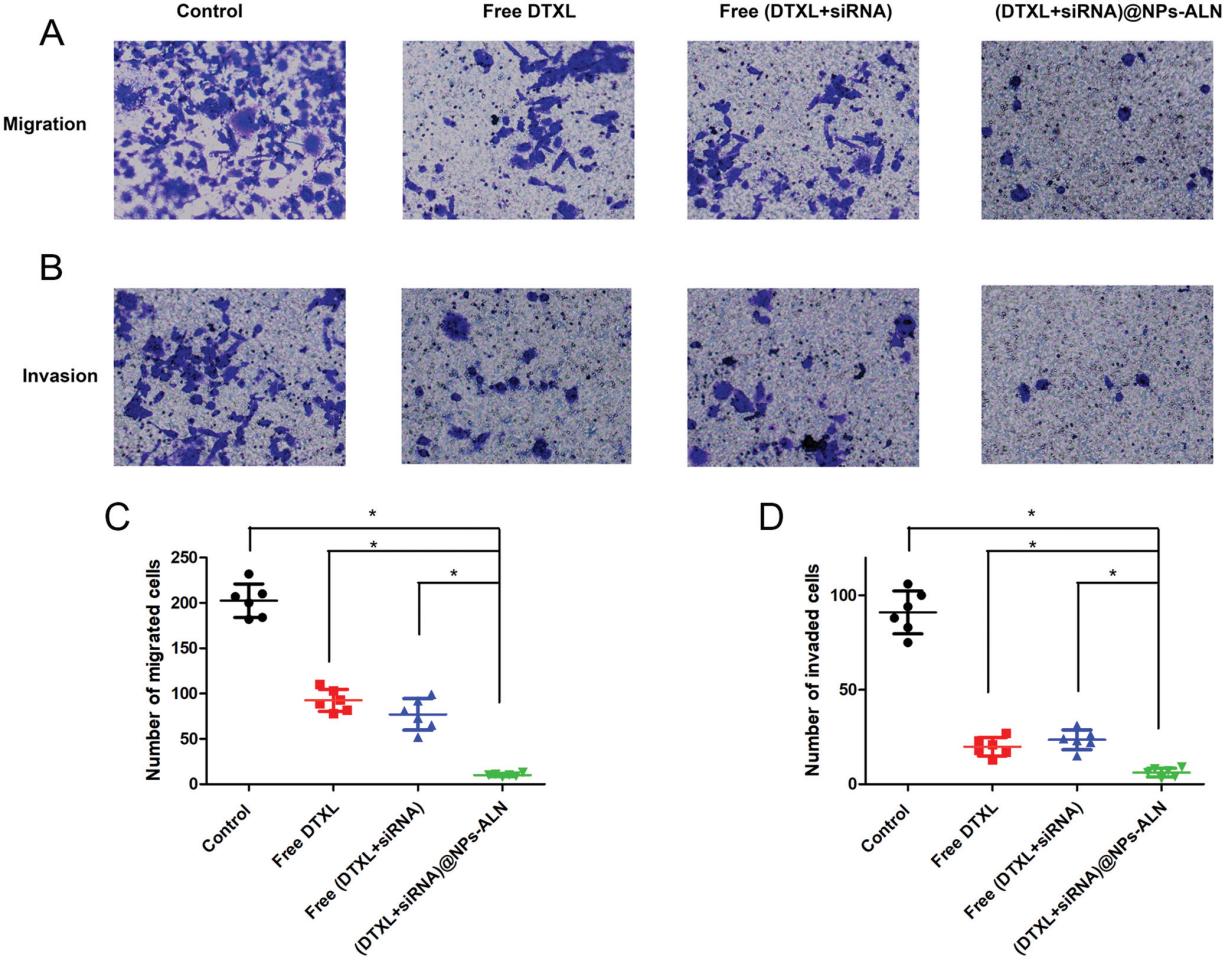
PC-3 cell migration (A) and invasion (B)were analyzed using transwell methods, (DTXL + siRNA)@NPs-ALN effectively inhibited cell migration and invasion. Statistics analysis was performed to analyze the number of migrated cells (C) or invaded cells (D) of different groups. Data were expressed as the mean ± *SD* (*n* = 3). *p < 0.05.

Since the migration and invasion of tumor cells are two important aspects of tumor cell metastasis, we studied the effects of drug-loaded NPs on tumor cell migration and invasion, respectively. The (DTXL + siRNA)@NPs-ALN effectively inhibited the tumor cell migration and invasion when compared with the other three groups (Figure 7).

### 3.8. Animal study

**Figure 8.**
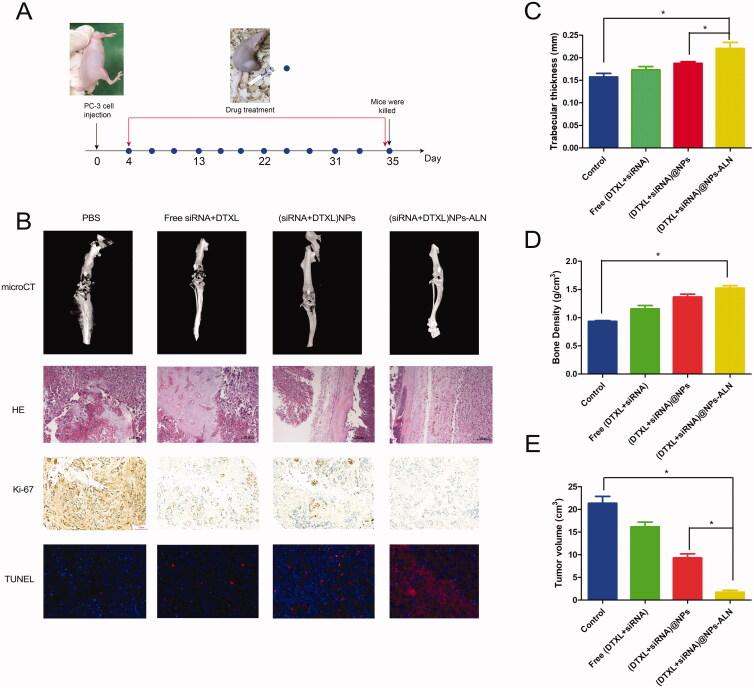
Anti-cancer activity of (DTXL + siRNA)@NPs-ALN for mice prostate cancer bone metastasis. (A) the time points for establishment of prostate cancer bone metastasis and treatment. (B) the microCT images, HE image of bone metastasis treated with different groups, and the Ki-67 expression level and tumor cell apoptosis were also evaluated using immunohistochemistry and TUNEL staining. (C) tibia trabecular thickness and bone density (D) were also evaluated, and the tumor volume was also evaluated when mice were sacrificed (E). Data were expressed as the mean ± *SD* (*n* = 6). *p < .05.

After successfully establishing a PCa bone metastasis model and verifying its successful establishment by HE staining and microCT imaging, we conducted a series of animal studies. First, nude mice bearing PCa bone metastasis were treated with DTXL or siRNA every other day (Figure 8(A)) and sacrificed after a period of 31-day treatment. The microCT imaging of the tumor-loaded tibias and femurs revealed very obvious bone destruction in the PBS group and free (DTXL + siRNA) group, while the (DTXL + siRNA)@NPs-ALN group showed less severe osteolytic bone lesions radiographically, indicating that (DTXL + siRNA)@NPs-ALN alleviated the bone damage lesion of the cancer-loaded tibia (Figure 8(B)). The H&E staining also indicated that cancer cells invaded and destructed the tibia normal bone structure of mice in the PBS group and free (DTXL + siRNA) group, while the tibia trabecular structure of mice was not damaged in the (DTXL + siRNA)@NPs-ALN group, which was consistent with the microCT results, reflecting that (DTXL + siRNA)@NPs-ALN could effectively inhibit PCa bone metastasis progression (Figure 8(B)). The tibia trabecular thickness and bone density decreased in the PBS group, free (DTXL + siRNA) group and (DTXL + siRNA)@NPs group, but did not decrease in the (DTXL + siRNA)@NPs-ALN (Figures 8(C,D)). Ki-67 staining of bone metastases revealed that the cancer cells in the (DTXL + siRNA)@NPs-ALN group showed the lowest proliferation capacity compared with the other three groups, indicating that (DTXL + siRNA)@NPs-ALN effectively inhibited cancer cell proliferation. Results of the TUNEL assay indicated that the apoptotic cancer cell rate was the highest in the (DTXL + siRNA)@NPs-ALN group compared with the other groups, suggesting that (DTXL + siRNA)@NPs-ALN effectively induced cancer cell apoptosis (Figure 8(B)). Additionally, measurement of the tumor volume after 31-day treatment, revealed that the mean tumor volume of the (DTXL + siRNA)@NPs-ALN group was 1.73 cm^3,^ which was much smaller than those of the PBS group (21.33 cm^3^), free (DTXL + siRNA) group (16.17 cm^3^) and (DTXL + siRNA)@NPs group (9.30 cm^3^) (Figure 8(E)).

### 3.9. NP toxicity evaluation

Moreover, the *in vivo* toxicity of free (DTXL + siRNA), (DTXL + siRNA)@NPs, and (DTXL + siRNA)@NPs-ALN was evaluated using histological analysis. The major organs morphology was observed user microscope, we found that there was no obvious damage in the heart, liver, spleen, lung, kidney in any of the four groups (Figure S2(A)), and the body weight of all mice increased steadily during the 31-day treatment (Figure S2(B)). These results indicated that (DTXL + siRNA)@NPs-ALN NPs had no obvious systemic toxicity.

## 4. Discussion

Although the mortality of PCa has significantly declined in recent years, the death rate of patients with PCa bone metastasis does not change remarkably (Siegel et al., 2020). Since the mechanism of PCa bone metastasis is not fully elucidated yet, as the interaction between PCa cell and bone microenvironment is complex, and the cancer cells in the bone microenvironment also lead to bone lesions, such as an osteolytic or osteogenic lesion (Cook et al., 2019; Ihle et al., 2019). The PCa bone metastasis remains very difficult to cure, so it is necessary to find novel treatment methods. In this study, we constructed a DDS comprised of bone targeting LCP NPs to co-deliver DTXL and SHH siRNA. The application of this DDS *in vivo* revealed that when the (DTXL + siRNA)@NPs-ALN reached the PCa bone metastasis site, both DTXL and siRNA were released to disturb the bone microenvironment and inhibit the PCa cell proliferation and migration, and finally inhibit the bone destruction caused by the cancer cell. The LCP NPs were prepared according to our previous methods (Zhang et al., 2019), these preparation methods effectively prevented CaP bulky aggregation, and DOPE phospholipid was used to stabilize the CaP cores of the NPs. Both DOPE-PEG-ALN and DOPE formed lipid bilayer spheres and dispersed evenly around the CaP core. The thermogravimetric analysis of NPs revealed the presence of a high proportion of lipid in the NPs. The PEG segment of the DOPE-PEG-ALN increased the circulation time of CaP NPs, and PEG formed a hydration shell which prevented NPs from interacting with blood components (Abe et al., 2015). The stability of NPs is very important for its further use, the coating-lipid enhanced the stability of our NPs, and our NPs did not aggregate at 37 °C or RT for as long as 72 h. The siRNA was condensed in the CaP core and DTXL was encapsulated in the asymmetric lipid-bilayer, and the encapsulation efficiency of both was above 80%. The (DTXL + siRNA)@NPs-ALN, in particular, showed a sustainable controlled release profile, which is a feature that would be favorable to achieve long-term antitumor effects.

The bisphosphonate ALN was used as a bone-targeting ligand that enabled the NPs to accumulate in the PCa bone metastasis, due to strong affinity of ALN for bone minerals, as revealed by previous studies showing that ALN-modified NP effectively accumulated in the bone metastasis, while non-modified NP did not (Chen et al., 2020; Long et al., 2021). Moreover, the ALN molecules shed from the NP could inhibit osteoclast activity and thus impair bone resorption, an effect that may alleviate the bone-related clinical symptom of PCa bone metastasis (Cummings et al., 2020). Remarkably, in this study, the ALN modified NPs were taken up much more efficiently than the non-modified NPs. However, the mechanism underlying this phenomenon remains elusive, and further studies are needed to fully elucidate it.

The bone microenvironment plays important role in the PCa bone metastasis progression, especially the paracrine SHH signaling (Wu et al., 2017; Furesi et al., 2021). This study showed that the SHH protein promoted PCa bone metastasis, which was consistent with the previous study that the SHH paracrine signaling was found to be involved in the regulation of cancer cells, osteoblasts, and osteoclasts (Wu et al., 2017). The co-culture system of PC-3cells with MC3T3-E1 osteoblasts used in this study revealed that co-culture promoted the secretion of SHH protein by PC-3 cells and this SHH protein bound to MC3T3-E1 cells and promoted the expression of SHH-related proteins, such as RANKL and IL-6, which were determined by ELISA. Additionally, the co-culture supernatant enhanced osteoclast differentiation and osteoblast mineralization. Importantly, the SHH protein was also found to interact with PC-3 cells through an autocrine pathway, which was verified by evaluating relevant protein changes in PC-3 cells. The SHH autocrine pathway has previously been reported to influence PCa proliferation (Sanchez et al., 2004; Bushman, 2016).

The *in vitro* antitumor mechanism was also studied, it was demonstrated that (DTXL + siRNA)@NPs-ALN exhibited combinational cancer cell killing effects, and effectively induced cytotoxicity, cell cycle arrest at G2/M phase, apoptosis, and autophagy, all these effects ultimately contributed to cancer cell death. The autophagy-related protein LCBII increased while the p62 protein decreased, which was consistent with the fluorescence analysis and TEM results, enhanced autophagy may lead to increased cell apoptosis. The evaluation of cell migration and invasion found that (DTXL + siRNA)@NPs-ALN effectively inhibited cancer cell migration and invasion abilities, which are very important for cancer cell metastasis (Jacob et al., 1999), suggesting that this may be one important reason for inhibiting PCa bone metastasis. The mice PCa bone metastasis model was successfully established by intraosseous inoculation of cancer cells according to previous studies (Dai et al., 2016), and was verified by microCT and H&E staining (DTXL + siRNA)@NPs-ALN effectively inhibited tumor growth and the bone destruction by the cancer cell, the trabecular thickness and bone density in this group were almost similar to those of the normal bone. This excellent therapeutic efficacy was likely due to several reasons, such as the good bone targeting ability of the ALN-modified NPs, the effective cancer cell killing effects of the (DTXL + siRNA)@NPs-ALN, and the osteoclast functions inhibition and osteoblast functions promotion, *etc*. The CaP NPs exhibited good biocompatibility, and this study also found by H&E staining that the major organs of the mice which were administered with NPs did not have damage. Additionally, the mouse body weight did not decrease during the 31-day treatment, both of these aspects reflected that the NPs were safe and non-toxic.

In conclusion, we prepared a DDS consisting of ALN-conjugated NPs to deliver DTXL and siRNA, and used it in a mouse PCa bone metastasis model to demonstrate its feasibility *in vivo*, besides evaluating its *in vitro* antitumor effects (DTXL + siRNA)@NPs-ALN showed improved bone-targeting capacity, inhibited osteoclast differentiation and promoted osteoblast differentiation mainly by affecting the SHH paracrine signaling in the bone microenvironment. This DDS represents a facile strategy to treat PCa bone metastasis and showed great potential for future translation into clinical practice.

## Supplementary Material

Supplemental Material
