# Supplementary figures and images for: Bone-targeted nanoplatform enables efficient modulation of bone tumor microenvironment for prostate cancer bone metastasis treatment
Source: Drug Deliv. 2022 Mar 14;29(1):889–905. doi: 10.1080/10717544.2022.2050845 (PMC8928789; doi:10.1080/10717544.2022.2050845)

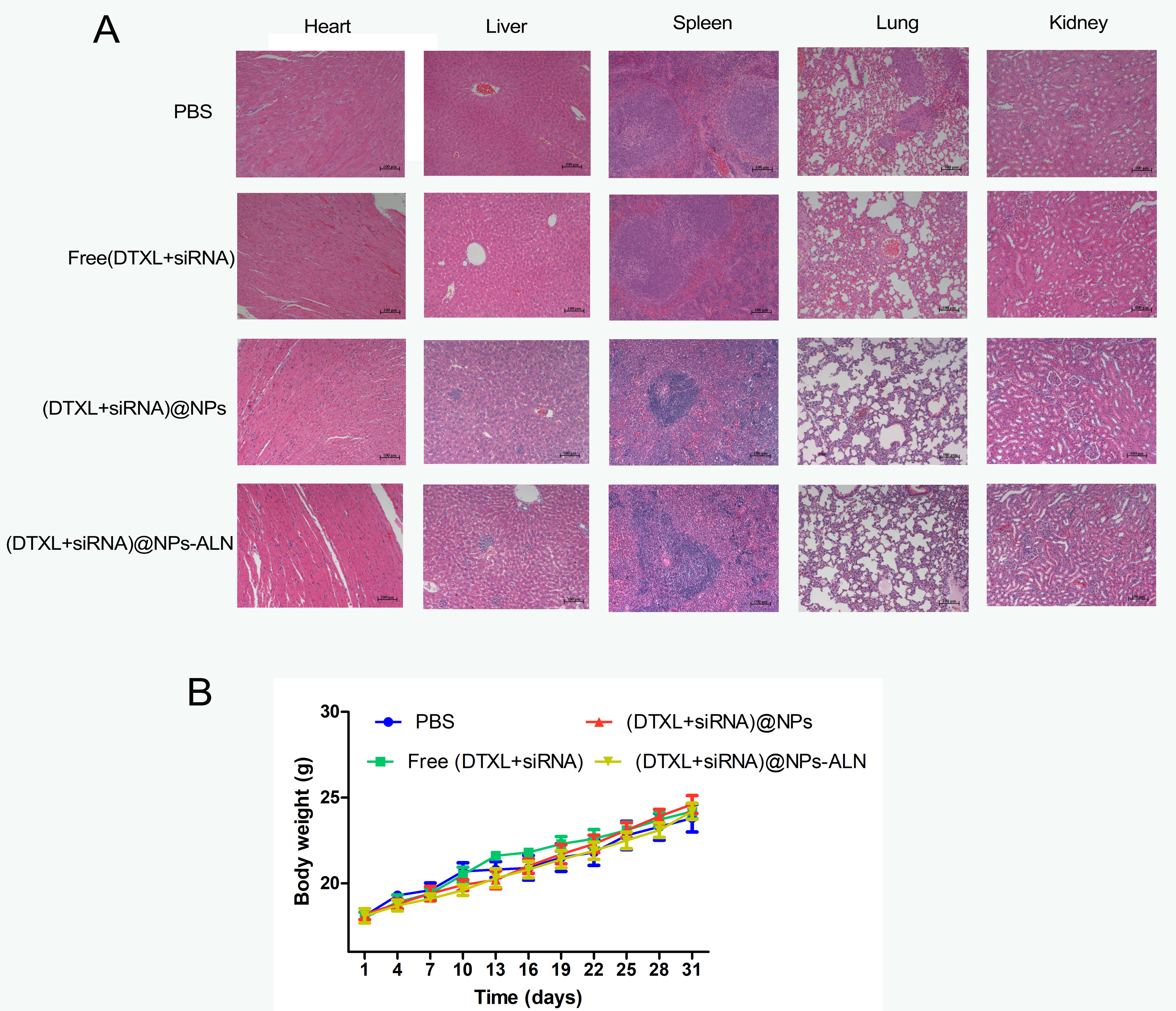

Supplement: Supplemental Material [file IDRD_A_2050845_SM9589.zip › Figure S2.tif]
